# Buried Treasure: Contradictions in the Perception and Reality of Women's Leadership

**DOI:** 10.3389/fpsyg.2021.684705

**Published:** 2021-05-26

**Authors:** Margaret M. Hopkins, Deborah Anne O'Neil, Diana Bilimoria, Alison Broadfoot

**Affiliations:** ^1^Management, University of Toledo, Toledo, OH, United States; ^2^Management, Bowling Green State University, Bowling Green, OH, United States; ^3^Organizational Behavior, Case Western Reserve University, Cleveland, OH, United States; ^4^San Diego Gas and Electric, San Diego, CA, United States

**Keywords:** gender, performance evaluation, women in leadership, leadership behaviors, gender bias

## Abstract

The impact of gender on assessments of leadership performance and leadership potential was examined through two clusters of leadership behaviors, one set related to traditional constructions of leadership labeled *directing others* and another associated with contemporary constructions of leadership labeled *engaging others*. Based on data collected from a sample of 91 senior leaders in one US financial services organization over a 3-year period prior to Covid-19, the results showed a negative relationship between *directing others* behaviors and leadership potential ratings for females and a positive relationship between these variables for males. A negative relationship between *engaging others* behaviors and performance assessments was also found for females. This study highlights the continuing bias in leadership assessments of women and explores the contradictions between the perception and the reality of women's leadership.

## Introduction

Organizations face a persistent gender gap in leadership. In the United States, women comprise nearly half of the labor force, yet only 6.2% of S&P 500 company's CEOs are women (Catalyst, 2021). Globally, women make up 46.9% of the workforce across all countries, yet one-third of global businesses have no women in senior management roles (Catalyst, 2017; Pew Research Center, 2017). On the contrary, men are twice as likely as women to reach the senior executive or CEO level (Thomas et al., 2017). A Wall Street Journal Executive Task Force for Women in the Economy (Barsh and Yee, 2012) measured 60 companies on four metrics of successful gender diversity. Eight companies met no metrics, while 52 companies met only one metric. These results are troubling indicators for women who hope to not only reach senior leadership positions but also realize their full potential in those roles.

The glass ceiling has shown few signs of cracking, since the number of women represented at the higher organizational levels has remained relatively consistent. A Catalyst (2012) study of more than 4,000 MBA graduates found that the starting salaries of women were less than their male counterparts across all industries. This investigation also discovered that men realized much more compensation through their promotions than women, 21 vs. 2%. Not only do women start their careers with a financial disadvantage, but the gender gap appears to widen over the course of their careers, and the global pandemic has only exacerbated this gap. As of 2020, women in formal employment earned only 79% of what men in formal employment earned globally in average monthly wages (International Labour Organization, 2020).

In the financial services industry, women are 50% of the entry-level positions, but a gender gap increases in the leadership pipeline from 42% of manager roles to 27% of senior vice presidents (Catalyst, 2020). In 2019, only 21.9% of senior leadership roles were held by women in financial services (Catalyst, 2020). Just 34% of senior-level women in the industry indicate that they have received career advancement advice from their senior leaders, while 44% of their male peers state they have (Chin et al., 2018). The financial services sector in North America has a 24% gap between men and women in their rates of promotions from entry-level to manager (Catalyst, 2020).

Factors that contribute to a persistent lack of females in top leadership positions have been documented, ranging from sex discrimination, and double standards to stereotyping of sex roles and leadership roles to bias in performance evaluations, and work-family conflicts (e.g., Joshi et al., 2015; Botelho and Abraham, 2017; Thomas et al., 2017; Padavic et al., 2020). At the same time as these challenges to women have been documented, there has also been an ongoing debate about whether women have certain advantages in leadership (Vecchio, 2002; Eagly, 2007) based on contemporary models of effective leadership. As opposed to a traditional view of leadership as authoritative and agentic, modern leadership perspectives emphasize teamwork and collaboration, interpersonal skills, and communal behaviors (e.g., Martin, 2006; Gilley et al., 2008). These relationally-oriented behaviors have been featured as optimal for leadership effectiveness, and they have also been directly linked in classic studies to women's ways of leading (Eagly, 1987; Rosener, 1990; Eagly and Karau, 2002). Thus, the argument has been made that these contemporary models of leadership offer certain advantages to women.

The purpose of this study is to investigate whether women leaders continue to face bias given the norms and expectations of how women should behave in senior leadership roles; or whether women realize advantages due to the alignment of their ascribed behaviors and more modern conceptualizations of effective leadership. While there appears to be a direct relationship between the current descriptors of effective leadership and the tendencies of women leaders, there is also evidence that there are few female advantages accruing to women in leadership positions. Our study examines the leadership behaviors of women and men in one financial services organization, and the impact of their behaviors on performance and potential assessments of their leadership.

## Theory and Hypotheses

### Effective Leadership Behaviors

Leadership has been a topic in organizational science research for almost a century. The Center for Creative Leadership conducted a global study to explore present and future definitions of leadership (Martin, 2006). According to 84.3 percent of responding leaders, the definition of effective leadership has shifted away from self-focused, heroic leadership to leadership that focuses on empowerment of others.

In addition to empowering others, collaborative skills such as building and sustaining relationships and participative management are recognized as being increasingly important for effective leadership (Powell and Graves, 2003; Martin, 2006). Teamwork and coaching have also been acknowledged as central elements of effectiveness (Eagly and Carli, 2003; Gilley et al., 2008). Leaders who are democratic in their style of leading received higher performance evaluations than those who tended toward an autocratic style of leading (Luthar, 1996). Fletcher (2004) defined what she labeled as post-heroic leadership with three characteristics: leadership is shared and distributed, leadership is relational, and the expected outcomes of leadership are growth and learning.

Thus, the composite picture of effective leadership places a strong emphasis on social skills. “…being a good manager …is less about competitiveness, aggression, and task orientation and more about good communication, coaching and people skills, and being intuitive and flexible, all more typically or at least stereotypically associated with women.” (Cooper and Lewis, 1999, p. 41).

### Perspectives of Female and Male Leadership

As women have assumed positions of leadership in organizations, considerable attention has been paid to the differentiating aspects of female and male leaders. This is an important area of inquiry because the behavior and leadership styles of a person directly relate to their performance and opportunities for advancement (Eagly et al., 2003). There are three predominant focal areas of investigation, and the results of studies in these areas not only vary but are contradictory.

The first set of research finds that there are no significant differences between the behaviors of male and female leaders (e.g., Nieva and Gutek, 1980; Bartol and Martin, 1986; van Engen et al., 2001). A second avenue of research suggests that feminine styles of leadership present certain advantages to women (e.g., Vecchio, 2002). A third collection of research studies concludes that there are indeed distinctions between male and female leaders related to stereotypes and other factors, which typically result in negative implications for female leaders (e.g., Lyness and Heilman, 2006; Scott and Brown, 2006; Eagly and Carli, 2007; Koch et al., 2015).

#### A Female Advantage

A number of studies have discussed the possibility of a female advantage. A McKinsey report (Desvaux and Devillard, 2008) identified that women, more so than men, use five of the top nine leadership behaviors that improve organizational performance. Another study found that women outperformed men in 28 of 31 skill areas, and these authors concluded that women were seen as more effective (Perrault and Irwin, 1996). A Business Week headline stated, “As leaders, women rule,” based on a study that found women executives received higher ratings on 42 of 52 skills measured and another study where women outranked men in 20 of 23 leadership areas (Sharpe, 2000). High-level women leaders were evaluated as both more agentic and more communal than their male counterparts; with the suggestion that once women break through the glass ceiling and are given credit for successful outcomes, they may have a distinct advantage over men (Rosette and Tost, 2010). A meta-analysis of 45 studies regarding transformational, transactional and laissez-faire leadership styles discovered that female leaders were more transformational than male leaders (Eagly et al., 2003). These results “…attest to the ability of women to perform very well in leadership roles in contemporary organizations” (Eagly and Carli, 2003, p. 583), as transformational leadership is directly related to leadership effectiveness (Bass, 1997; Judge and Piccolo, 2004). “As employees' implicit theories and organizations' explicit models of effective leadership continue to integrate charismatic leadership behaviors that demand strong social and emotional skills, women may have a decided advantage regarding employment selection and promotion decisions for leadership roles and developmental opportunities.” (Groves, 2005, p. 43).

#### Disadvantages for Females

Another stream of research takes a different standpoint and describes how female leaders face greater hurdles than their male counterparts. A study Pew Research Center (2015) found that men and women believe that female leaders are equally qualified as their male peers, yet two-thirds of the respondents believe that it is easier for men to reach senior-level positions. Primary barriers to leadership for women have been reported as: inadequate management of leadership pipeline; little, if any, targeted development to grow women's leadership capabilities; lack of role models; lack of gender diversity awareness among management; and lack of appreciation for women's expertise (Cooke et al., 2016). Women report that their skills are not recognized by their organizations (Rutherford, 2001), that they face additional obstacles to their career advancement and are less satisfied with their career opportunities as compared to men (Lyness and Thompson, 1997). Establishing their credibility is a common theme as they struggle with higher performance standards and lower rewards (Van Velsor and Hughes, 1990; Catalyst, 2007). A meta-analysis of sex differences spanning 30 years found that the sex differences in rewards were 14 times greater than the sex differences in performance evaluations (Joshi et al., 2015). These authors concluded that women often close the gap in performance but not the gap in rewards such as salary and promotions. Lammers and Gast (2017) found that claims of a female leadership advantage may unwittingly sustain gender inequality by undermining support for affirmative action to reduce female underrepresentation.

Women report that having help from above is their number one career success factor, while men note that having a track record is a primary reason for their success (Morrison et al., 1992). Yet highly visible developmental assignments are not given to women as often as they are to men (Lyness and Thompson, 2000) which results in women being placed at a distinct disadvantage. Women consistently report being excluded from networks, especially informal networks, as another barrier to their advancement (Ragins et al., 1998; Lyness and Thompson, 2000). Sometimes even formal organizational networks designed to increase women's opportunities fail to break through the perceptions of women as diversifying organizations but not being treated as valued business partners (O'Neil et al., 2011). A Catalyst study of 4,000 high potential women and men found that more women than men have mentors, yet women are less likely to advance in their careers (Carter and Silva, 2010). One conclusion from this finding is that women are over-mentored and under-sponsored compared to their male peers (Ibarra et al., 2010). Sponsoring is defined as going beyond giving advice and actually advocating for individuals with senior leaders (Ibarra et al., 2010).

Stereotypes of men and women have remained relatively consistent, with women being perceived as more communal in behavior and men more agentic (Eagly, 1987; Powell et al., 2002). In one study of several hundred corporate leaders, both men and women indicated that women show “take care” behaviors while men show “take charge” behaviors, indicating a gender-stereotypical perspective (Catalyst, 2005). Implicit leadership theories are relatively stable mental models that are formed early in life (Schyns, 2006). Prior studies report that both men and women describe managers as having predominant masculine characteristics typically associated with males (Powell et al., 2002; Schein, 2007). “Leadership has traditionally been construed as a masculine enterprise with special challenges and pitfalls for women” (Carli and Eagly, 2001, p. 633). A ‘think manager, think male” stereotype persists in organizations (Schein, 2007). In an examination of gender bias toward female leaders, more individuals indicated a preference to work for male leaders than female leaders (Elsesser and Lever, 2011). The primary reasons given for favoring a male leader were focused on negative attitudes toward female leaders or better chemistry with men, while the foremost reasons in favor of working for a female leader were positive aspects such as her compassion and competence.

Role incongruity presents another challenge for women. Agentic behaviors, the predominantly masculine orientation, are the expected behaviors for leaders (Eagly and Karau, 1991). Yet communal behaviors are those expected of women based on their gender role (Eagly and Karau, 2002). Thus, leaders are expected to show agentic behaviors and women are expected to show communal behaviors. This role incongruity presents a double bind for women: act in the expected leader role and women are seen as too tough; act in the expected gender role and women are seen as too soft (Catalyst, 2007). When women behave in ways that are assertive and decisive, they are viewed as competent, yet they are not viewed as interpersonally effective as those women who use stereotypical feminine behaviors (Catalyst, 2007). One study found that the behavior of female and male senior executives was more similar than different, thus concluding that the expectations of their job role took primacy over the expectations of their gender role at the highest organizational levels (Lyons and McArthur, 2007). However, their similar behaviors did not result in similar treatment as interview participants talked about leadership when asked to discuss male leaders while gender was a topic raised when asked to discuss female leaders (Lyons and McArthur, 2007).

There is a cost for women due to the incongruity between leadership role and gender role expectations (Koch et al., 2015). Both descriptive and prescriptive gender stereotypes can lead to bias in evaluations of women leaders (Heilman, 2001). Descriptive refers to differences in how men and women actually behave while prescriptive denotes norms about how they should behave. Descriptive bias occurs when women leaders are stereotyped as having less leadership potential than men, while prescriptive bias happens when female leaders are evaluated less favorably than men due to the belief that leadership is a masculine enterprise. Heilman and Martell (1986) found that the primary reason for systematic bias in performance assessments was the evaluator relied on stereotypes at the time of the rating as opposed to identifying the individual's actual performance. When an expectation about performance exists, the evaluator will tend to rate the behaviors that are consistent with their beliefs and mental models (Bauer and Baltes, 2002).

Two predominant theoretical frameworks exist for considering gender bias in performance appraisals: the stereotype fit model and the relational demography approach (Roberson et al., 2007). The stereotype fit model (Heilman, 1983) indicates that evaluators compare their stereotype of the person being rated with another stereotype of their perceived requirements of the role. Thus, when women are being evaluated, stereotypes about their gender role conflict with stereotypes about the leadership role and there is a perceived lack of fit between the two roles. Heilman (1983) argued that gender bias increases when there is a perceived lack of fit. For example, autocratic behavior used by female leaders was evaluated more negatively than the same behavior in male leaders, while comparable evaluations were given for democratic behavior (Eagly et al., 1992). Relational demography examines the demographic similarities or differences between the evaluator and the person being evaluated and proposes that less favorable evaluations occur when there are dissimilarities (Tsui and O'Reilly, 1989; Tsui and Gutek, 1999). One study illustrating both the stereotype fit and the relational demography frameworks found that women tended to be evaluated as less effective than their male counterparts when they were in male-dominated roles and roles perceived as masculine, and when there were more men performing the ratings (Eagly et al., 1995). This study also reported that when the leader role was defined in feminine terms, the women were favored in their effectiveness ratings.

In summary, there is a strong body of research which provides evidence for the existence of a pro-male bias in the evaluation of performance. Agentic behavior is perceived as less advantageous for women due to role incongruity, and women have received less favorable evaluations as a result (Eagly et al., 2003). Decision makers, who are predominantly male, have been more likely to select men than women for leadership roles (Heilman, 1995) and to evaluate male leaders more favorably than females demonstrating equivalent performance (Hopkins and Bilimoria, 2008; Joshi et al., 2015). Another account found that promoted females had even higher performance ratings than their promoted male counterparts, indicating that the women had to exceed the performance of the men in order to be considered for promotion (Lyness and Heilman, 2006).

Based on these arguments, we propose:

*H1) Gender will moderate the relationship between leadership behaviors associated with directing others and performance evaluations. The relationship for males will be positive and for females will be negative*.

Leadership behaviors related to engaging others effectively, e.g., teamwork, adaptability, and empathy, are behaviors distinctly characteristic of women. Arguments have been made that there is a female advantage, and that women may in fact benefit from demonstrating collaborative behaviors (Vecchio, 2002; Eagly and Carli, 2003). Women are more effective when they operate within their gender-congruent role (Eagly et al., 1995). While it has been proposed that there may be a female advantage, this proposition has yet to be affirmed in empirical work. Effective male leaders have strengths in both masculine and feminine behaviors (Wrolstad et al., 1992; Heilman et al., 1995); and men have been found to have more flexibility to demonstrate both stereotypically masculine and feminine behaviors, this being viewed as an enhancement to their leadership (Eagly and Johannesen-Schmidt, 2001). We hypothesize that there will be a benefit in the performance ratings for both men and women when they demonstrate leadership behaviors related to engaging others.

*H2) Gender will not moderate the relationship between leadership behaviors associated with engaging others and performance evaluations. Both males and females will show a positive relationship*.

Distinct from performance appraisals, evaluations of leadership potential assess the future capabilities of individuals for promotion into senior organizational positions. Evaluation bias against women occurs not only in the assessment of women's actual and perceived behaviors, illustrating descriptive and prescriptive gender stereotypes, but also in the ratings of the potential of women for higher-level leadership roles (Eagly and Karau, 2002). According to one global study (Mattis, 2001) male managers gave feedback on job performance to both male and female direct reports. However, the male managers spent time discussing career paths and future advancement opportunities only with the male employees. The focus of the manager-female employee conversations was on their present performance while the manager-male employee discussions were centered on future potential in addition to present performance. Disparate levels of performance required of males and females in order to be considered for promotion have been reported (Lyness and Heilman, 2006). A study examining men and women in one organization found that despite women's exceptional performance on organizational measures, the women did not receive superior ratings on their overall leadership potential nor did they advance at a faster rate than the men in the organization (Shore, 1992). In order to be on the high potential list for promotions, females need to outperform their male counterparts (Morrison et al., 1992). Evaluations of potential for leadership in the future are based at least in part on assessments of a candidate's perceived riskiness for leadership (van Esch et al., 2018). In this study, gender moderated the relationship between qualifications and selection for senior leadership, in that moderately qualified women were seen as riskier for senior leadership than moderately qualified men (van Esch et al., 2018). Although there is ample extant research on the evaluation of leadership performance between men and women, there are fewer studies examining the differences in potential ratings. The results of existing studies suggest gender-based bias in the evaluation of leadership potential and a higher perceived riskiness of women for leadership. We propose to examine the differentials in potential ratings between men and women based on a novel approach: their demonstration of certain leadership behaviors–either directing others or engaging others' behaviors:

*H3) Gender will moderate the relationship between leadership behaviors associated with directing others and high potential ratings. The relationship for males will be positive and for females will be negative*.*H4) Gender will moderate the relationship between leadership behaviors associated with engaging others and high potential ratings. The relationship for males will be positive and for females will be negative*.

## Methods

### Sample

The sample consisted of 91 senior leaders, 26 females (29%) and 65 males (71%), in one financial services institution with offices throughout the United States. This institution is one of the Top 100 US Best Banks named by Forbes magazine. The respondents mirrored the gender proportions of the senior leadership population in this organization, which comprised 130 individuals, 40 females and 90 males. The sample group identified as either male or female, and no participant responded to the category option of “Other.” Over a 2-year period, five groups of the organization's top leaders participated in a custom-designed executive development program conducted by a local university focused on developing leadership capabilities linked to organizational business priorities. Their selection was based solely on their leadership level, and all senior leaders attended the program. Our sample was drawn from this population. One organization as the research site controlled for possible contextual differences in the criterion variables of performance and potential for male and female leaders. A comparison of males and females in one organization ensured that any observed gender differences were not due to factors such as differences in industries or management hierarchies across organizations.

The mean age of the participants was 46 for the males and 44 for the females. On average, the males had been in managerial roles for 17 years and the females for 14 years, and had held their current positions an average of 2.6 and 3.0 years, respectively. Both the men and women had been with the organization an average of 14 years.

### Data Collection

An introductory email was sent from the Executive Vice President of Human Resources to the sample population, explaining the study and inviting participation. The researchers then emailed the population to describe the study and the method of participation in greater detail. The data were collected prior to the Covid-19 pandemic.

### Measurement of Variables

#### Independent Variable: Leadership Behaviors

The leadership behaviors were assessed through a multi-rater assessment instrument composed of behavioral indicators designed to measure leadership competencies (Boyatzis et al., 2001, 2002). Sample items from the 72-item instrument include “in a group, encourages others' participation” for the Teamwork and Collaboration competency and “articulates a compelling vision” for the Inspirational Leadership competency. Respondents were asked to complete each item on a five-point frequency scale ranging from “Never” to “Consistently.” The instrument is supported by evidence of reliability and validity, and the leadership competencies have demonstrated desirable psychometric properties (Sala, 2002; Boyatzis and Sala, 2004).

The 360-degree leadership assessment was completed by each participant leader, their manager, peers, direct reports, clients, and others as pre-work prior to entering the executive development program. Participants' leadership competency scores from the other assessors on this multi-rater instrument, not their self-ratings, were used. An average of twelve other raters' feedback reports were received for each individual leader, with a total of 1,092 others' leadership assessments examined.

To arrive at one score per item per person, an average of the total others' item scores for each individual competency for each of the study's participants was computed. To obtain a per person competency score for the individual competencies, an average of each competency's item scores was calculated. Empirical research suggests that the aggregated scores of other rater groups are advantageous in order to reduce random error and perceptual differences among the observations by others (Atwater and Yammarino, 1992) as the respondents tend to focus on different aspects of the leader's competencies (Hogan et al., 1994; Salam et al., 1997). Thus, the ratings of other people tend to provide a more complete picture of a leader's behaviors.

Boyatzis and Sala (2004) ran an exploratory factor analysis using an oblique rotation in order to explore the scale structure of the instrument. The first factor loaded with the following competencies: Emotional Self Awareness, Accurate Self Assessment, Transparency, Empathy, Developing Others, and Teamwork and Collaboration. The Conflict Management competency had two of four items loading on this first factor. The second factor included: Self Confidence, Achievement, Inspirational Leadership, and Change Catalyst. Adaptability and Initiative each had one-half of their items loading on the second factor.

Similar to Boyatzis and Sala (2004), we ran an exploratory factor analysis (EFA) to investigate the factor structure of the competency scales. A principle axis EFA with an oblique rotation was conducted. We chose to use an oblique rotation as Boyatzis and Sala (2004) found some of the competencies loaded onto both factors. Although this was an EFA, we constricted the number of factors to two in an attempt to confirm Boyatzis and Sala (2004) scale structure; thus, this analysis was somewhat confirmatory in nature.

Table 1 contains the factor loadings from this analysis. The competencies appear to load strongly on only one of the two factors, aside from the Developing Others competency. Looking at the competencies that loaded strongly onto factor 1, they appeared to be measuring the construct we labeled as *engaging others*. These competencies include: Adaptability, Empathy, Conflict Management, Teamwork & Collaboration, Emotional Self Awareness, Accurate Self Assessment, and Transparency. Examining the competencies that loaded strongly onto factor 2, they appeared to be measuring the construct we labeled as *directing others*. These competencies include: Achievement Orientation, Initiative, Inspirational Leadership, Change Catalyst, and Self Confidence. Since the competency, Developing Others, loaded fairly equally yet not necessarily very strongly onto either factor, that competency was eliminated from the data analysis. Therefore, seven competencies were used to measure the scale of *engaging others* and five competencies were used to measure the scale of *directing others*. These two factors are similar to the factors observed by Boyatzis and Sala (2004), providing us with confidence in these scales. The internal consistency reliability for *directing others* was 0.86 and for *engaging others* was 0.89. These reliabilities were calculated using the average item score at the competency level. For example, for the *directing others* measure, five competencies were used, but the scores for each of these five competencies were measured by more than one item. However, the items measuring each competency were averaged together to get a total score for that competency. Then the total score for the five competencies were used to evaluate measure reliability.

**Table 1:** Exploratory factor analysis for the leadership competencies scale–rotated factor matrix.

	**Factor**	
**Leadership competencies**	**Engaging others**	**Directing others**
Self-Confidence	−0.107	**0.751**
Achievement orientation	0.277	**0.760**
Initiative	0.363	**0.577**
Inspirational leadership	0.462	**0.784**
Change catalyst	0.065	**0.753**
Developing others	0.506	0.477
Adaptability	**0.676**	0.321
Emotional self awareness	**0.679**	0.229
Accurate self-assessment	**0.799**	0.079
Transparency	**0.645**	0.163
Empathy	**0.779**	0.104
Conflict management	**0.623**	0.133
Teamwork & collaboration	**0.831**	0.073

*Extraction Method: Principal Axis Factoring*.

*Rotation Method: Equamax with Kaiser Normalization*.

*Rotation converged in 3 iterations*.

#### Dependent Variables: Performance and Leadership Potential

Organizational measures of performance and potential for every individual in the sample group were collected for a 3 year period: the current year of the study and the prior 2 years. Annual performance ratings on a three-item scale (1 = development needed; 2 = full performance; 3 = exceptional performance) and annual potential ratings on a three-item scale (1 = mastery; 2 = growth; 3 = turn) were conducted for every leader in the organization by the leader's immediate manager. These assessments were standard annual evaluations used in the organization. All of the assessing managers to whom the participants reported were male. The scores were averaged over 3 years to compute two outcome numbers representing the participant's measure of performance and leadership potential.

#### Moderating Variable: Gender

Gender was coded as zero for males and one for females.

### Data Analysis

To test Hypotheses 1–4, two hierarchical moderated regression analyses were conducted; one analysis for the dependent variable *performance* (for H1 & H2) and a second analysis for the dependent variable *potential* (for H3 & H4). We included *age* and *years within a management role* at the first step in both moderated regression analyses to control for possible confounds in the relationships. In the second step the main effects, which included the variables *gender, engaging others*, and *directing others*, were entered into the regression equation. Finally, in the third step, the interaction terms were added; specifically, the interaction between *gender* and *engaging others* and the interaction between *gender* and *directing others*. All variables were centered to help control for multicollinearity and to aid in the interpretation of results (Aiken and West, 1991).

## Results

Means, standard deviations, reliabilities (when applicable), and inter-correlations for the study's variables are included in Table 2. In addition to gender and age, we tested the significance of marital status, parental status, and education level and found no effects.

**Table 2:** Descriptive statistics and correlations.

**Means, standard deviations, reliabilities, and intercorrelations among study variables**									
	**Variables**	***M***	***SD***	**1**	**2**	**3**	**4**	**5**	**6**
1	Performance	2.31	0.46	(0.51)					
2	Potential	2.11	0.53	0.188	(0.68)				
3	Directing others	4.02	0.25	0.228^*^	0.280^**^	(0.86)			
4	Engaging others	3.87	0.26	0.123	0.091	0.556^*^	(0.89)		
5	Age	45.43	5.65	−0.234^*^	−0.426^**^	−0.191	0.007		
6	Years in management role	16.1	6.76	−0.121	−0.340^**^	−0.144	0.059	0.709^**^	

*N = 91. Reliabilities are listed in parentheses along the diagonal when appropriate*.

(*) *Correlations significant at the 0.05 level*.

(**) *Correlations significant at the 0.01 Level *.

The results from the two hierarchical moderated regression analyses can be found in Table 3.

**Table 3:** Results of moderated regression analyses.

**Steps**	**Variables**	**Performance^a^**	**Potential^a^**
**Step 1 (controls)**			
	Age	−0.316^*^	−0.386^**^
	Years in a management role	0.116	−0.057
	*R^2^*	0.061	0.183
	*F*_(2, 87)_	2.840	9.723^**^
**Step 2 (main effects)**			
	Directing others	0.155	0.198
	Engaging others	0.010	−0.036
	Gender^b^	0.011	0.024
	*R^2^*	0.086	0.214
	*F*_(5, 84)_	1.576	4.565^**^
**Step 3 (moderating effects)**			
	Directing others × Gender	−0.180	−0.363^**^
	Engaging others × Gender	−0.248^*^	0.111
	*R^2^*	0.191	0.279
	*F*_(7, 82)_	2.765^*^	4.540^**^

*N = 91*.

a *Standardized beta coefficients*.

b *0, male; 1, female*.

* *p < 0.05;*

** *p < 0.01*.

### Moderated Regression Results for Performance

**Figure 1:**
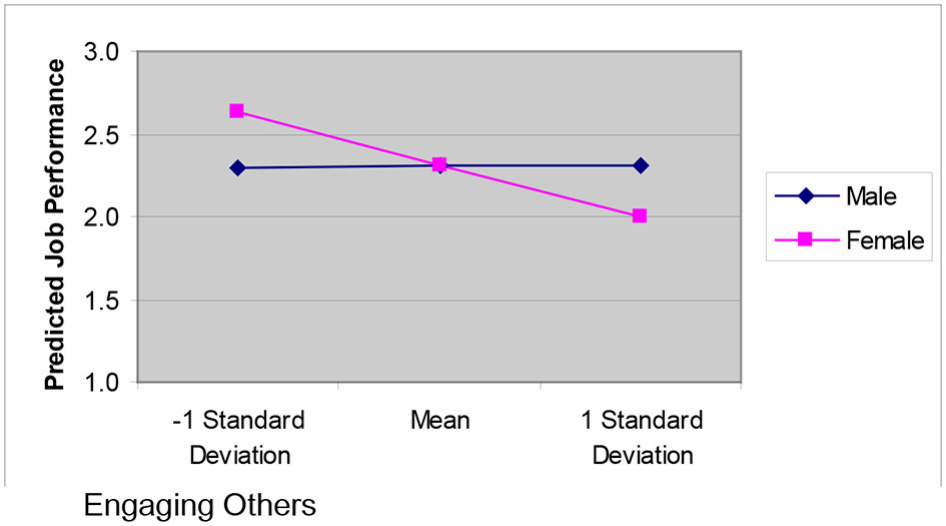
Interactive effects of the engaging others leadership competencies on performance.

For the moderated regression with *performance* as the dependent variable, only the results from the third step can be examined because only at this step is the model significant [*F*_(7, 82)_ = 2.765, *p* = 0.012]. Nineteen percent of the variation in *performance* scores is accounted for by the variables in the third step, which includes the interaction terms. Looking within this step, the interaction between *gender* and the competency cluster *engaging others* is significant (*B* = −0.248, *p* = 0.049). To further understand this interaction, we did a *post-hoc* moderator analysis as outlined in Aiken and West (1991). Figure 1 charts the simple slopes predicting *performance* from *engaging others* broken down by *gender*.

As can be seen, for males there is no relationship between *performance* and *engaging others*; however, for females there is a strong negative relationship between these two variables. These results fail to support Hypothesis 2. The observed negative relationship for *engaging others* and *performance* for females runs completely counter to Hypothesis 2. In addition, we failed to support Hypothesis 1 as the interaction between *directing others* and *gender* was not significant and we could not look at the main effects in step 2 as the model for step 2 was not significant.

### Moderated Regression Results for Potential

**Figure 2:**
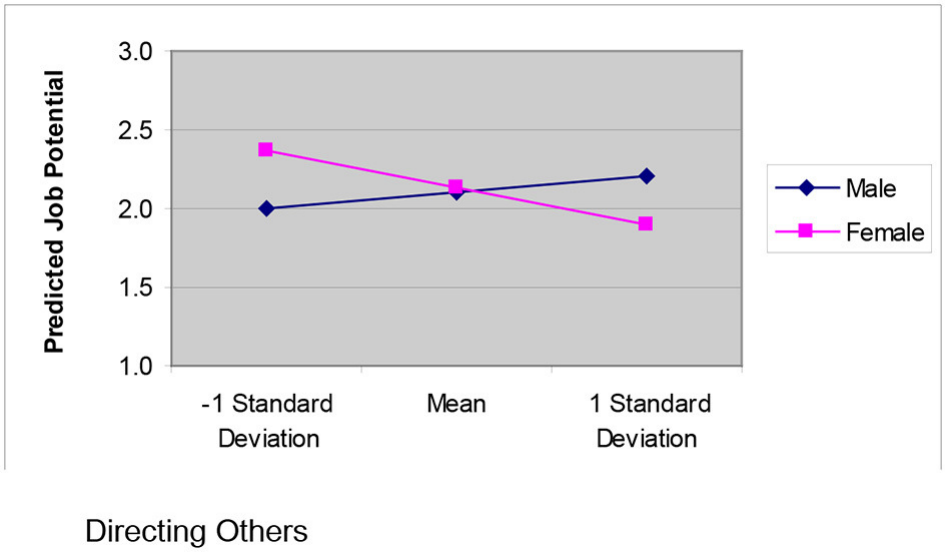
Interactive effects of the directing others leadership competencies on potential.

As Table 3 indicates, all three steps in the moderated regression analyses were significant. Looking within step 1 [*F*_(2, 87)_ = 9.723, *p* = 0.000], the control variable *age* is significantly and negatively related to *potential* (*B* = −0.386, *p* = 0.006). This finding makes sense as a younger employee would have more time for growth and the potential to be successful in the future, whereas an older employee is less likely to be perceived as having as much potential for future improvement. Eighteen percent of the variation in *potential* scores is accounted for by age and years in a managerial role. The second step, adding the main effects, was also significant [*F*_(5, 84)_ = 4.565, *p* = 0.001]; however, none of the main effects were significantly related to the dependent variable, *potential*. Twenty one percent of the variation in *potential* scores is accounted for by the variables in the second step. Finally, the third step, which includes the interaction terms, was significant [*F*_(7, 82)_ = 4.540, *p* = 0.000]. Twenty eight percent of the variation in *potential* scores is accounted for by the variables in the third step, which includes the interaction terms. Within this third step, the interaction between the competency cluster *directing others* and *gender* was significant (*B* = −0.363, *p* = 0.008). To further understand this interaction, we did a *post-hoc* moderator analysis as outlined in Aiken and West (1991). Figure 2 charts the simple slopes predicting *potential* from *directing others* broken down by *gender*.

As can be seen, for males there is a positive relationship between *potential* and *directing others* whereas for females there is a negative relationship between these two variables. This provides full support for Hypothesis 3. Considering *potential*, the interaction between *engaging others* and *gender* was not significant; therefore we failed to find support for Hypothesis 4.

## Discussion

This study highlights the continuing tension between the perceptions vs. the reality of women's leadership. While a common belief is that women excel in skills and behaviors related to effective leadership, as the French saying goes “the more things change the more they stay the same.” The slow rate of career advancement for women in the financial services industry will likely continue at a snail's pace, given the results of this study. Women in our study continue to face significant inequities in the assessment of their leadership performance and their leadership potential. Gender bias in the perceptions of leadership remains a persistent hurdle for women to overcome; and the resulting differential impact on women's careers will likely be exacerbated as a result of the Covid-19 pandemic which has resulted in many women stepping away from the workforce. Whether women demonstrated people-oriented, relational skills such as those valued for contemporary leadership or whether they exhibited achievement-oriented behaviors, there was a negative impact on their leadership assessments.

Leadership behaviors related to *engaging others* had no positive relationship with assessments of performance or leadership potential for both the female and the male leaders. This finding is counterintuitive to the existing literature regarding effective leadership. The predominant, contemporary conception of leadership incorporates a kinder, gentler leader prototype, one who develops relationships with others and works collaboratively to achieve results. If these behaviors have been identified as integral to effective leadership, then why are they not being acknowledged as such in organizational leadership assessments? Perhaps this result indicates that the research in this area is ahead of organizational practice.

A widespread belief is that women tend to rely on the interpersonal aspects of leadership with an emphasis on participation and teamwork. A previous study has labeled the relational practice of women in organizations as “disappearing acts” (Fletcher, 1999) whereby their relational behaviors often “get disappeared,” not because they are ineffective but because they are associated with the feminine or softer leadership skills that are stereotypically expected of women. Not only did we find that these leadership behaviors are not necessarily acknowledged as important to effective leadership, but we also discovered that these behaviors are devalued when women demonstrate them. In this study, women were in fact penalized in their performance assessments when they demonstrated the relational-oriented behaviors of *engaging others*. One explanation might be that these behaviors are solely attributed to women's gender roles and not their leadership roles resulting in role incongruity. This result supports Heilman's (1983) stereotype fit theory by highlighting the disconnect between the expected leadership role behaviors and the expected gender role behaviors. Persistent stereotypes of the leader role being a predominantly masculine enterprise may be another explanation for this troubling finding (c.f., Schein, 2007).

The *directing others* behaviors (e.g., achievement orientation and change catalyst) in our study were found to be negatively related to leadership potential assessments for women and positively related to leadership potential assessments for men. It would appear in this case that the prescriptive gender role behaviors for women trump the leader role behaviors for women, and consequently women pay a price. Women demonstrating out-of-gender role behaviors such as achievement orientation or driving change conflict with the stereotype fit model for women. As Schein (2007) think manager, think male studies illustrate, there is a common notion that men are naturally presumed to have leadership potential. “Of course Jack can take this on, we know he can do it.” A similar assumption is not made for women, however, and our results underscore this belief. “Well, Jacqueline cannot do it because we have never seen her do it before.” An Atlantic magazine article entitled “Why Women Still Can't Have It All” (Slaughter, 2012) caused a great deal of debate over this question. The author states “If women are ever to achieve real equality as leaders, then we have to stop accepting male behavior and male choices as the default and the ideal.” (p.6).

Previous empirical studies of bias against female leaders have produced mixed results. Investigations in laboratory settings using hypothetical scenarios have tended to reveal bias against female leaders (e.g., Eagly et al., 1992). Arguments have been made that studies using actual leaders in the workplace will result in little or no bias against female leaders (e.g., Eagly et al., 1995). The conclusion drawn from this latter research is that familiarity with an individual will reduce stereotyping since the assessments are being made based on actual interactions and experiences. Our findings do not support this viewpoint. Our study examined the leadership performance and potential assessments of male and female leaders by their all-male direct supervisors in senior leadership roles. Whether the female leaders were demonstrating stereotypical masculine behaviors or stereotypical feminine behaviors, there was a negative impact on their leadership performance and potential appraisals.

Entrenched archetypes that define leadership as a masculine enterprise remain in spite of data that relates feminine behaviors to effective leadership (e.g., Scott and Brown, 2006; Badura et al., 2018). The assumption that women have certain advantages given these relationships was not supported in our study; in fact, we discovered just the opposite. Although women excel in the contemporary definition of effective leadership behaviors, there continues to be gender bias in the assessments of women in leadership roles. The prescriptive gender role appears to take precedence over the leadership role when evaluating female leaders. Women leaders are primarily viewed through their gender role and assessed as to whether their behaviors match this role. When women exhibit typical leadership role behaviors such as achievement orientation and change catalyst, they pay a price in their leadership potential assessments. When women exhibit typical gender role behaviors such as teamwork and empathy, they also pay a price in their leadership performance assessments. We suggest that this outcome is due to women not being considered strong or agentic enough to be effective leaders.

Based on our results we have to conclude that female behaviors are not valued in performance appraisals or in assessing leadership potential. This is a perceptual problem, not one based on actual behaviors or reality. Notions about how women should behave persist, resulting in prescriptive bias for women leaders. We continue to discover differences in the experiences of women leaders which often result in overwhelming obstacles. While there is an abundance of empirical research identifying effective leadership with behaviors that are stereotypically female (e.g., Eagly et al., 2003; Offermann and Foley, 2020), we find no evidence of acknowledging this in practice. The process that organizations use to assess their leaders has not appeared to change to reflect this new reality. Not only should leadership assessment instruments be examined for possible bias, but also the methods by which individuals conduct assessments of women leaders in particular should be reviewed for inherent bias. The reality persists that the majority of those individual who are conducting these assessments are men. Prior research has demonstrated that gender stereotypes can lead to bias in the evaluations of women (e.g., Heilman, 2001). Perceptions and prescriptions about female leadership behavior obscure and devalue women's actual accomplishments. A commitment to examine existing archetypes and mental models of female leaders with an aim of replacing them with verifiable behaviors and actions is warranted.

Finally, comprehensive remedies to address the attitudinal and structural issues are required. In conjunction with reviewing individual practices, investigating organizational policies, procedures and structures will help to determine how they may be contributing to impediments for women in leadership roles. Identifying solutions of a structural, political and cultural nature as recommended in prior research may provide a productive path forward (Fagenson, 1990; O'Neil et al., 2008).

### Limitations of the Study

While a single financial services organization controlled for organizational culture and context, this may also limit the generalizability of the findings. Additional organizational factors that may have contributed to the results, such as possible company policies with regard to promoting women, were not examined. The relatively small sample of women leaders in the study is another potential limitation, although the respondents mirrored the organization's population of male and female leaders. The lack of a more robust sample of women leaders unfortunately reflects the larger issue of the dearth of women in senior organizational leadership. In addition, while the sample mirrored the pre-dominantly Caucasian, male, and female population of similar ages in the organization's leadership, this also limits the generalizability. The issue of measurement equivalence between ratings of males and females, and the possibility of a different reference point that might have been used in evaluating them, might also be considered. Measurement equivalence suggests that while there might have been a quantitative difference in the interpretation of what a score on an item means for the genders, there could also have been qualitative differences as well. Due to the sample size, this could not be examined.

### Implications for Future Research

Additional assessments of male and female leadership behaviors should be conducted with validated 360-degree instruments in order to expand our understanding of the real or imagined differences between men and women. A composite picture of a leader's behaviors, as opposed to relying solely upon self-reports or the perspectives of a single constituency group such as direct reports or managers, provides a more complete picture of their impact on others. In addition, analyses comparing the leadership behaviors between the individual rater groups' scores (i.e., manager, peers, direct reports, clients, and customers' scores) can provide a deeper understanding of the importance of demonstrating specific behaviors to each of these distinct groups.

Examinations of the impact of leadership behaviors on assessments of performance and leadership potential with larger samples of male and female leaders from multiple organizations will extend this research and the generalizability of the results. It will be important to address the question of whether the practice of relying on perceptions and assessing female leaders with prescriptive bias is a universal practice in organizations or was it unique to the organization in this research study.

### Implications for Practice

First and foremost, it is imperative that organizational decision-makers recognize the overt and covert, as well as the individual and structural biases that prevent women from assuming leadership roles and reaching their full leadership potential. Empirical evidence of these biases abounds (Morrison et al., 1992; Stroh et al., 1992; Powell et al., 2002; Schein, 2007), yet there has been limited progress in dismantling the barriers to women's advances. Organizational leaders must be proactive in uncovering and diminishing existing biases.

Beyond continuing to discover the unrecognized and unacknowledged potential of female leaders, organizations need to examine their practices, procedures, and policies on a regular basis to determine whether they are reinforcing gender stereotypes and stereotypical behavior. For example, hiring procedures, training and development opportunities, benefits packages, leave policies, and performance, salary and promotional evaluations can all play a part in contributing to gender stereotypes. One recommendation for performance evaluations would be to include 360-degree assessment tools in the formal assessment process for leaders, to include data from a variety of perspectives and individuals. Leaders in policy-making positions should periodically reflect on their own behaviors, not only for their own learning and development but also to see whether they are behaving in ways that contribute to gender stereotypical practices. Perhaps they are sending subtle messages throughout the organization that sustain and support such behaviors.

The repertoire of leadership behaviors used in organizational assessments for hiring, training and development, and promotions must be expanded to reflect best practices and include the broadest possible range. Leaders who have the capacity to demonstrate a large repertoire of behaviors are able to effectively respond to a variety of situations. Organizational systems that have a limited framework for essential leadership behaviors will restrict their ability to recruit and develop outstanding leaders.

Leadership behaviors focused on relationship management, teamwork, collaboration, and empathy must be acknowledged as important for organizational viability, growth and change when demonstrated by both males and females. These behaviors provide the necessary bond for an organization's continued development. It is not enough to rely solely on leadership behaviors that are germane to one's gender, particularly if they might be discredited, or to behaviors that strictly match one's organizational position and are incongruent with gender. What is needed is the recognition of a broad array of leadership behaviors demonstrated by both women and men.

Special attention needs to be paid to the opportunities for, and pace of, career advancement for women. Career paths for women in particular must be reconsidered to reflect boundaryless (Arthur, 1994) or kaleidoscope (Mainiero and Sullivan, 2005) career perspectives as well as the more traditional linear or hierarchical models. Organizational leaders should expand their notions of careers and career paths to assist in better understanding, motivating, and rewarding all employees. Finally, individual leaders have an obligation to speak up when they observe gender stereotypes in organizations. Silence perpetuates the practice of seeing men and women through narrow lenses, limiting the potential contributions of all organizational members.

## Data Availability Statement

The raw data supporting the conclusions of this article will be made available by the authors, without undue reservation.

## Ethics Statement

The studies involving human participants were reviewed and approved by Case Western Reserve University. The patients/participants provided their written informed consent to participate in this study.

## Author Contributions

All authors listed have made a substantial, direct and intellectual contribution to the work, and approved it for publication.

## Conflict of Interest

The authors declare that the research was conducted in the absence of any commercial or financial relationships that could be construed as a potential conflict of interest.
